# Metabolomic Signatures for the Effects of Weight Loss Interventions on Severe Obesity in Children and Adolescents

**DOI:** 10.3390/metabo12010027

**Published:** 2021-12-30

**Authors:** Min-Ji Sohn, Woori Chae, Jae-Sung Ko, Joo-Youn Cho, Ji-Eun Kim, Ji-Yeob Choi, Han-Byul Jang, Hye-Ja Lee, Sang-Ick Park, Kyung-Hee Park, Peter J. van der Spek, Jin-Soo Moon

**Affiliations:** 1 Department of Pediatrics, Seoul National University College of Medicine and Children’s Hospital, Seoul 03080, Korea; minnn31@gmail.com (M.-J.S.); kojs@snu.ac.kr (J.-S.K.); 2 Department of Pediatrics, Dongguk University Ilsan Medical Center, Goyang 10326, Korea; 3 Department of Clinical Pharmacology and Therapeutics, Seoul National University College of Medicine and Hospital, Seoul 03080, Korea; yunus@snu.ac.kr (W.C.); joocho@snu.ac.kr (J.-Y.C.); 4 Department of Biomedical Sciences, Seoul National University Graduate School, Seoul 03080, Korea; jejemint@snu.ac.kr (J.-E.K.); miso77@snu.ac.kr (J.-Y.C.); 5 Department of Preventive Medicine, Seoul National University College of Medicine, Seoul 03080, Korea; 6 Cancer Research Institute, Seoul National University, Seoul 03080, Korea; 7 Division of Endocrine and Kidney Disease Research, Department of Chronic Disease Convergence Research, Korea National Institute of Health, Cheongju 28159, Korea; greatstar@korea.kr (H.-B.J.); hyejalee@korea.kr (H.-J.L.); sooin0108@korea.kr (S.-I.P.); 8 Department of Family Medicine, Hallym University, Anyang 14068, Korea; beloved920@gmail.com; 9 Department of Pathology and Clinical Bioinformatics, Erasmus University Medical Center Rotterdam, 3000 CA Rotterdam, The Netherlands; p.vanderspek@erasmusmc.nl

**Keywords:** biomarkers, metabolomics, obesity, pediatric, child, interventions

## Abstract

Childhood obesity has increased worldwide, and many clinical and public interventions have attempted to reduce morbidity. We aimed to determine the metabolomic signatures associated with weight control interventions in children with obesity. Forty children from the “Intervention for Children and Adolescent Obesity via Activity and Nutrition (ICAAN)” cohort were selected according to intervention responses. Based on changes in body mass index z-scores, 20 were responders and the remaining non-responders. Their serum metabolites were quantitatively analyzed using capillary electrophoresis time-of-flight mass spectrometry at baseline and after 6 and 18 months of intervention. After 18 months of intervention, the metabolite cluster changes in the responders and non-responders showed a difference on the heatmap, but significant metabolites were not clear. However, regardless of the responses, 13 and 49 metabolites were significant in the group of children with obesity intervention at 6 months and 18 months post-intervention compared to baseline. In addition, the top five metabolic pathways (D-glutamine and D-glutamate metabolism; arginine biosynthesis; alanine, aspartate, and glutamate metabolism; TCA cycle (tricarboxylic acid cycle); valine, leucine, and isoleucine biosynthesis) including several amino acids in the metabolites of obese children after 18 months were significantly changed. Our study showed significantly different metabolomic profiles based on time post obesity-related intervention. Through this study, we can better understand and predict childhood obesity through metabolite analysis and monitoring.

## 1. Introduction

The prevalence of childhood obesity has increased worldwide [1]. In some countries, obesity rates in children and adolescents exceed 30%. According to the Korean Ministry of Education, the Korean childhood obesity rate in 2018 reached 25%, increasing rapidly from 8.4% in 2008 [2]. Childhood and adolescent obesity are related to anthropometric and metabolic changes, such as metabolic syndromes, including dyslipidemia, hypertension, and insulin resistance [3]. Adolescent obesity is associated with several cancers and cardiovascular diseases in adulthood [4,5]. Anthropometric measurements, such as the body mass index (BMI) or waist–hip ratio, and circulating biomarkers, such as insulin or adiponectin, are currently used as general “obesity biomarkers” to identify disease risk due to obesity [6]. Most government authorities worldwide face difficulties in increasing the budget for obesity control, even though early intervention in childhood obesity is a well-known method effective for controlling the obesity epidemic. There is an unmet need to identify the most effective methods to control childhood obesity, monitor treatment effects before and after intervention, and select responsive candidates for treatment.

Obesity is caused by various complex factors, of which genetic and epigenetic factors are the major causes. These factors affect the lipidome, metabolome, and proteome, and various omics studies have investigated the causes of obesity [7]. Knowing the cause and risk factors of obesity is important not only for understanding the pathogenesis but also for finding effective personalized treatments. Metabolites are small molecules, substrates, intermediates, and end-products of cellular regulatory processes that play important roles in cellular and physiological energetics, structure, and signaling [8]. Metabotyping, in which individuals with similar metabolite patterns are grouped into clusters, plays a key role in the development and delivery of personalized nutrition [9]. Therefore, metabotyping of patients with obesity will play an essential role in developing a new understanding of and treatment methods for obesity.

As obesity rates increase, metabolic studies that reveal metabolomic signatures of patients with obesity and study metabolic changes such as inflammation or oxidative stress associated with obesity are increasing [10]. However, few studies have investigated childhood obesity, especially those involving weight-loss interventions [11,12,13,14,15]. A study on prepubertal children with obesity reported that urine trimethylamine N-oxide levels decreased after lifestyle intervention. Of the 32 identified metabolites, xanthosine, 3-hydroxyisovalerate, and dimethylglycine were altered following the intervention [16]. After an 8-week exercise program, the urine concentrations of pantothenic acid, glyceric acid, l-ascorbic acid, xanthine, and adenosine were higher in overweight adolescents than in the normal weight group [17]. Thus, long-term metabolite changes associated with weight intervention in obese children are needed to understand and treat obesity.

This study analyzed metabolites at different intervention intervals by selecting responders and non-responders and identifying metabolites that were significantly different. We also identified significant metabolite changes associated with long-term childhood obesity weight intervention. In addition, we checked whether clinical indicators have significant results with these metabolite changes. The metabolomic information from this study can be used to identify biomarkers related to childhood obesity and to develop future personalized treatments

## 2. Results

### 2.1. Selection of Study Population and Their Demographic Characteristics

Of the 242 patients of the ICAAN cohort, we finally included 40 obese patients in this study. In detail, 163 and 111 were followed up at 6 and 18 months after the weight control intervention, respectively. A total of 131 participants dropped out during the intervention, mainly because of busy schedules, no response, lack of willingness, or busy schedule of parents.

The clinical characteristics of the study population are presented in Table 1 and Figure S1. No significant differences were found according to sex, age, type of intervention between the groups, aspartate transaminase (AST), alanine aminotransferase (ALT), triglyceride (TG), high-density lipoprotein cholesterol (HDL cholesterol), low-density lipoprotein (LDL cholesterol), or triglyceride-to-HDL-cholesterol (TG/HDL) ratio at baseline or at 18 months post-intervention.

### 2.2. Profiles of Circulating Metabolites according to Responsiveness and Duration of Weight-Loss Intervention

**Figure 1:**
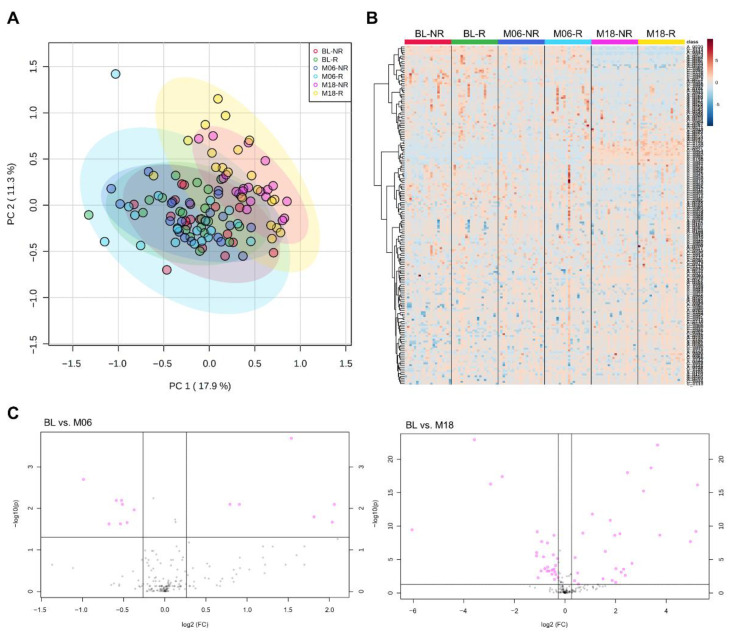
Plasma metabolome profiles of the study population. (**A**) Principal component analysis and (**B**) hierarchical cluster analysis showing time-dependent metabolic changes by weight-loss interventions rather than responsiveness to the intervention. (**C**) Volcano plots of significant metabolites (FDR adjusted *p*-value < 0.05, fold change > 1.2) at 6 months and 18 months post-intervention compared to baseline.

A total of 194 plasma metabolites were successfully identified from capillary electrophoresis time-of-flight mass spectrometry (CE-TOFMS) measurement on the basis of Human Metabolome Technologies (HMT)’s standard library and Known-Unknown peak library. First, we investigated metabolomic profiles of the study population according to responsiveness (Figure 1). A scores plot of principal component analysis (Figure 1A) and volcano plots (Figure S2) showed that no significant differences between responders and non-responders were found at any timepoints (baseline, 6 months, and 18 months post-intervention). However, interestingly, distinct metabolic profiles of 18 months post-intervention compared to baseline or 6 months post-intervention were observed, regardless of responsiveness to the intervention. These remarkable changes were clearly revealed by hierarchical clusters of metabolites on the heatmap (Figure 1B) and volcano plots (Figure 1C). Thirteen and forty-nine metabolites were significant (FDR adjusted *p*-value < 0.05, fold change > 1.2) at 6 months and 18 months post-intervention compared to baseline, respectively (Figure 1C, pink circles). Among them, 12 metabolites were significant at both timepoints: asparagine, glycerophosphocholine, N-acetyllysine, glutamine, O-acetylcarnitine, 8-hydroxyoctanoic acid, glucosamine, caproic acid, arginine, kyotorphin, N-acetylornithine, and prostaglandin F2a. A list of significantly changed metabolites by weight-loss intervention is represented in Table S1. These results suggest that long-term weight-loss intervention itself may induce metabolic changes, independent of effect of intervention (i.e., BMI z-score changes) in children and adolescents.

### 2.3. Weight-Loss-Intervention-Induced Changes in Metabolite Sets and Metabolic Pathways

**Figure 2:**
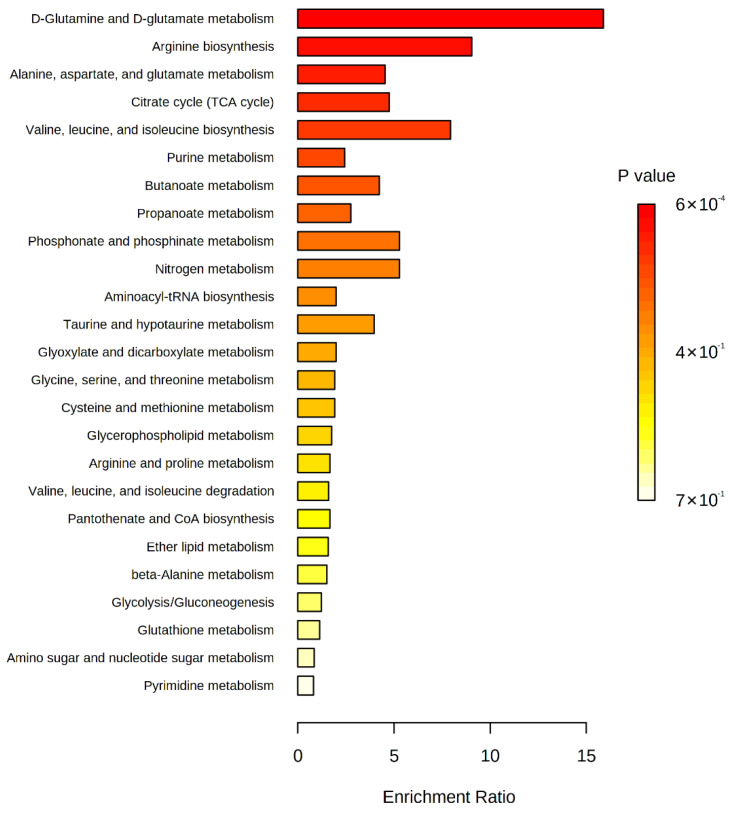
Enriched metabolite sets of significant metabolites between baseline and 18 months post-intervention on the basis of KEGG database.

**Figure 3:**
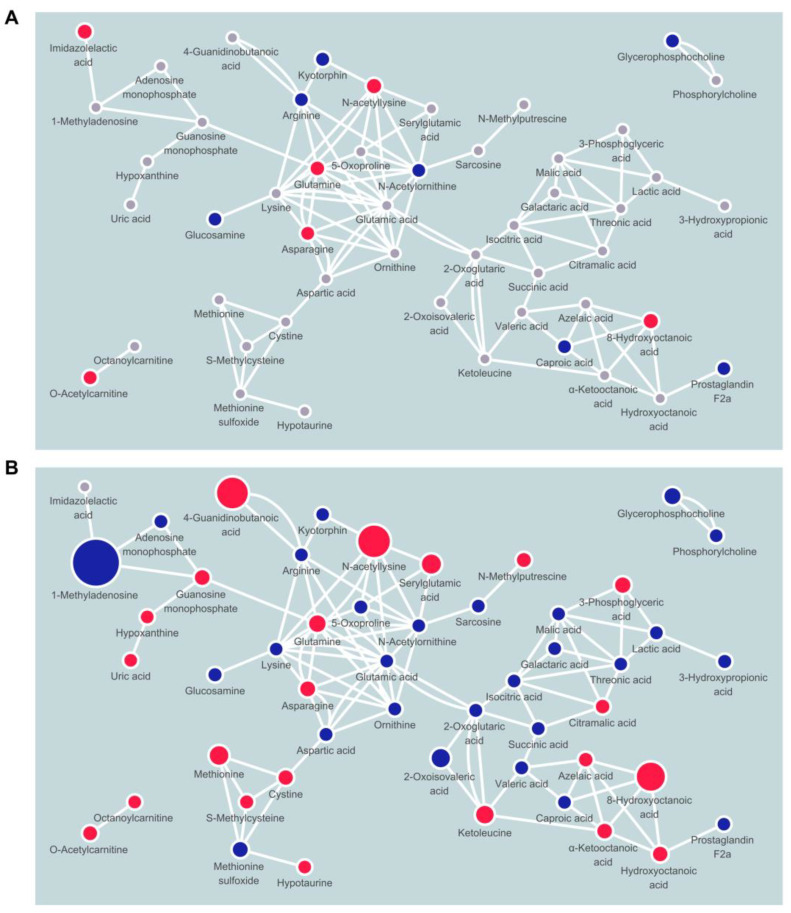
Network mapping showing significantly changed metabolic pathways by weight-loss intervention after (**A**) 6 months and (**B**) 18 months. Node color indicates direction of changes (red, up; blue, down) or non-significance (gray).

To understand which metabolic pathways are altered by long-term intervention, we performed metabolite set enrichment analysis with the significant metabolites between baseline and 18 months post-intervention (Figure 2 and Table S2). D-glutamine and D-glutamate metabolism and arginine biosynthesis were significantly modified (enrichment ratio > 9.0, FDR adjusted *p* < 0.05), and other metabolite sets including alanine, aspartate, and glutamate metabolism, tricarboxylic acid (TCA) cycle, and valine, leucine, and isoleucine biosynthesis were also enriched (enrichment ratio > 4.5, raw *p* < 0.05). We also constructed chemical and biochemical networks using these significant metabolites (Figure 3). Compared to metabolic changes in 6 months post-intervention (Figure 3A), carnitines and many organic acids including o-acetyl carnitine, octanoylcarnitine, azelaic acid, hydroxyoctanoic acid, and alpha-ketooctanoic acid were upregulated whereas galactaric acid, threonic acid, caproic acid, and alpha-ketoisovaleric acid levels were downregulated in the 18 months post-intervention group (Figure 3B). TCA cycle intermediates such as succinic acid, oxoglutaric acid, isocitric acid, malic acid, and phosphocholines were also significantly downregulated in the 18 months post-intervention group. In addition, levels of metabolites related to the methionine–cysteine pathway (cystine, methionine, s-methylcysteine, and methionine sulfoxide), urea-cycle-related metabolites, and amino acids (arginine, ornithine, N-acetylornithine, lysine, N-acetyllysine, 5-oxoproline, glutamine, glutamic acid) were also significantly changed.

## 3. Discussion

**Figure 4:**
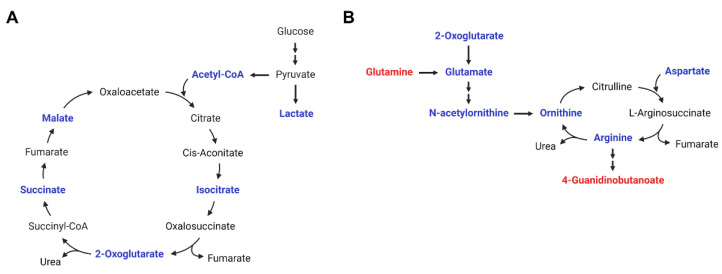
Metabolic pathway changes by weight-loss intervention. (**A**) TCA cycle and (**B**) arginine biosynthesis and urea cycle. (Red, metabolites increased in plasma at 18 months post-intervention compared to at baseline; blue, metabolites decreased in plasma at 18 months post-intervention compared to at baseline).

Previous studies have mainly described metabolites or genomes associated with childhood obesity or the BMI in comparison to control subjects [10,18,19,20]. We further analyzed metabolites showing significant differences according to weight control intervention responses in pediatric patients with severe obesity. However, metabolic differences by intervention response were not clear. Metabolites showing differences by the time of intervention were very diverse; however, we identified significant metabolic pathway changes in weight intervention in childhood obesity, regardless of the intervention type or response (Figure 4).

The metabolic pathways are included in the urea and TCA cycles and several amino acid (i.e., glutamine, glutamate, arginine, cysteine, and methionine) metabolic pathways. Several studies have already shown that glutamate is increased in obese children and glutamine is decreased in obese patients without weight interventions [12,21]. These previous results are consistent with our result that plasma glutamine was increased after weight-loss intervention, whereas glutamate was decreased. In particular, glutamate was found to be the metabolite with the highest bivariate correlation with body fat and insulin sensitivity in an American-Indian adolescent with obesity study [22].

Obesity is essentially a change due to an inflammation mechanism, and thus changes in various immune cells are affected. In obese and diabetic patients, changes in macrophage polarization can induce decreased α-ketoglutarate production and increased succinate in the TCA cycle, and glutamine metabolism is essential for the process [23]. Succinate, an intermediate product of the TCA cycle, is released from adipose tissue in obesity and plays an important role in inducing inflammation by macrophage activation [24]. Consequently, macrophage polarization involving glutamine metabolism and the TCA cycle in obese and diabetic patients might play a key role in obesity pathology. In this respect, our study showed that levels of TCA intermediates including isocitrate, malate, and oxoglutarate (α-ketoglutarate) as well as succinate were decreased by the weight-loss intervention, which implies anti-inflammatory effects of the intervention by minimizing polarization of macrophages.

Arginine, aspartate, and ornithine are associated with urea cycle metabolism and play an important role in ammonia detoxification. Our study showed that decreased urea cycle intermediates including aspartate, ornithine, and arginine in plasma, which implies downregulated production of ammonia by weight-loss intervention. Aspartate, like pyruvic acid, is an amino acid associated with the TCA cycle. The metabolic shift in pyruvic acid decreased after weight control intervention in overweight pre-adolescent and obese women [17,25]. Zheng et al. [26] reported amino acid profile changes from the POUND LOST and DIRECT trials, which confirmed metabolite changes after a 2-year diet-induced weight-loss intervention in adult patients with obesity. In both trials, weight loss was directly related to the concurrent reduction of branched-chain amino acids (BCAAs; leucine and isoleucine), aromatic amino acids (tyrosine and phenylalanine), and other amino acids (alanine, sarcosine, hydroxyproline, and methionine) [26]. Glutamine and glutamate were positively correlated with a homeostatic model assessment of insulin resistance (HOMA-IR) in an overweight-to-obese, dyslipidemic adult study, too [27].

Dysregulation of these various metabolic pathways and insulin resistance are associated with metabolic changes in childhood obesity, which may also be affected by pubertal development and the gut microbiome. During the pubertal stage, insulin sensitivity decreases by 50% and is associated with increased total body lipolysis and decreased glucose oxidation [28]. These alterations may have affected metabolic changes in our study population; previous studies have shown that metabolomic profiles change owing to increased adiposity measures in post-pubertal male groups [29]. The microbiota contributes to amino acid biosynthesis; thus, the same foods may contribute to different caloric and nutrient bioavailability in different individuals [30]. Gut microbial profiling of individuals with insulin resistance and insulin sensitivity is associated with different host dietary intervention responses and weight changes. In addition, studies in a mouse model suggest that the gut microbiome can activate BCAA synthesis pathways in obesity [31]. In our study, we did not consider these points, and this should be considered together in further research.

Taken together, these metabolic changes by weight-loss intervention might become phenotypic characteristics of metabolically healthy obesity (MHO). Some obese patients show a healthier phenotype than other obese patients, which is often observed in young and physically active patients with good nutritional status [32]. Although many clinical and behavioral characteristics of MHO such as anthropometric and clinical parameters, lifestyle factors, and comorbidities have been suggested, no concept of MHO is universally accepted yet [33]. However, MHO can be an ancillary criterion to redeem a BMI-based single definition by reflecting characteristics of obesity in various aspects. The metabolic changes we observed suggested that the weight-loss intervention can ameliorate pediatric patients toward metabolically healthy status. It also might be supported by individual clinical changes including AST, ALT, TG, HDL cholesterol, LDL cholesterol, and TG/HDL ratio. However, they were not statistically significant. One possible explanation is poor compliance of participants due to long-period intervention.

This study was limited by the availability of subjects for longitudinal studies owing to many dropouts from the childhood obesity intervention cohort. In addition, there was a possibility of selection bias in the results. Lower family functioning, exercise group, lower initial attendance rate, and non-self-referral pathways were significantly associated with early dropouts, and lower family functioning and lower initial attendance rates were associated with late dropouts in our cohort study [34]. Thus, it is important to focus on these factors to reduce the dropout rate in further intervention-based cohort research. In fact, poor family function was associated with high levels of depressive symptoms in the childhood obesity cohort [35]. Further research is needed to determine how these factors affect the intervention outcomes in children. In addition, this study did not analyze the factors related to adolescent age while observing changes in long-term follow-up during the intervention. In the heatmap, metabolic changes were most pronounced after 18 months, but the probability that hormonal changes and other factors affected the metabolites was not considered. Another limitation is that the patients’ hospital visit time and participation level were not considered in the study. In addition, factors such as diet could not be limited to homogeneity, which may have influenced the metabolite results. In particular, metabolites are affected by sampling time or what is consumed at the time of sampling; therefore, it is important to control these factors and follow the trend of metabolites.

In conclusion, weight-loss intervention induced serial changes in specific amino acid metabolites and a metabolic pathway regardless of response to interventions in childhood obesity. Long-term follow-up results of weight intervention and metabolite changes are more important than responses to weight-loss interventions, which may inform clear mechanisms of obesity. This is why it is important to regularly visit a clinic for treatment of obesity, and tracking changes in metabolites will be helpful in monitoring the individual. Therefore, this study can be utilized for the development of metabolite biomarkers related to childhood obesity treatment through a long-term follow-up study. It is also expected to contribute to large-scale research for the prediction and prevention of personal childhood obesity using metabolites in the future.

## 4. Materials and Methods

### 4.1. Study Population

We selected 40 obese subjects (BMI > 97th percentile for age and sex) from 242 patients in the ICAAN study cohort based on intervention responses. This 24-month post-intervention follow-up study was designed as a multidisciplinary intervention test to prevent excessive weight gain and to improve several health indices in children and adolescents (aged 6–17 years) with obesity in Korea [36,37,38]. Patients with obesity-related hereditary diseases or other underlying diseases were excluded. We observed follow-up data at baseline, 6 months, and 18 months post-intervention to observe the intervention effect trends. They were randomly divided into 3 groups and received interventions, including the usual care, exercise, and nutrition feedback group. Each group had a similar portion (usual care group (*n* = 84, 34.7%; exercise group (*n* = 74, 30.6%); nutrition feedback group (*n* = 84, 34.7%)), and all groups received five category interventions (nutrition, physical activity, group activity, parental education, and self-monitoring). The exercise group included the contents of the usual care group and added weekly exercise class and activity feedback. The nutrition feedback group received additional individual nutrition feedback including the usual care group contents.

Participants were categorized into two groups based on changes in BMI z-scores after 18 months. Twenty subjects presenting significant intervention effects were assigned to the target (responder) group and those with minimal weight loss to the non-responder group (*n* = 20). In the responder and non-responder groups, the changes in BMI z-scores were <−0.45 and >−0.1 in the responder and non-responder groups, respectively. Random sampling was not possible owing to the limited sample size; therefore, the portion and number of samples in each intervention group were considered. As no patients gained weight because of the intervention, patients with the least weight change were selected as the non-responder group based on total responders. A flowchart of the participant selection is provided in Supplementary Data (Figure S1). Anthropometric and laboratory assessment data and blood samples were collected at baseline and 6 and 18 months post-intervention in both groups, resulting in a total of 120 collected samples.

### 4.2. Sample Preparation and CE-TOFMS Analysis

The 120 plasma samples were transported from Seoul National University (SNU) to HMT via Young-In Frontier Co., Ltd. The samples were stored in a deep freezer below −80 °C. To each 50 μL of sample, 200 μL of methanol containing internal standards (L-methionine sulfone and D-camphor-10-sulfonic acid, 20 μM) was added, diluted with 150 μL of distilled water and mixed thoroughly. Each mixture (300 μL) was filtered through a 5-kDa cut-off filter (Ultrafree-MC-PHCC, HMT, Yamagata, Japan) to remove macromolecules. The filtrate was centrifugally concentrated and resuspended in 50 μL of distilled water immediately before analysis. The compounds were measured in the cation and anion modes using an Agilent CE-TOFMS system (Agilent Technologies Inc., Santa Clara, CA, USA) and a fused silica capillary i.d. 50 μm × 80 cm. Cationic metabolites were analyzed with a fused silica capillary (50 µm i.d. × 80 cm total length), with Cation Buffer Solution (Human Metabolome Technologies, solution ID: H3301-1001) as the electrolyte. The sample was injected at a pressure of 50 mbar for 5 s. The applied voltage was set at 30 kV. Electrospray ionization-mass spectrometry (ESI-MS) was conducted in the positive ion mode, and the capillary voltage was set at 4000 V. The spectrometer was scanned from *m*/*z* 50 to 1000. Anionic metabolites were analyzed with a fused silica capillary (50 µm i.d. × 80 cm total length), with Anion Buffer Solution (Human Metabolome Technologies, solution ID: I3302-1023) as the electrolyte. The sample was injected at a pressure of 50 mbar for 10 s. The applied voltage was set at 30 kV. ESI-MS was conducted in the negative ion mode, and the capillary voltage was set at 3500 V. The spectrometer was scanned from *m*/*z* 50 to 1000. To guarantee analytical reproducibility, we monitored the relative standard deviation of internal standards which are added in the sample (less than 10%).

### 4.3. Metabolomic Data Processing

Peaks detected by CE-TOFMS analysis were extracted using an automatic integration software (MasterHands ver. 2.17.4.19, Keio University; Tokyo, Japan) to obtain peak information, including *m*/*z* values, migration time (MT), and peak area. The peak area was then converted and normalized to a relative peak area by dividing by sample amount and internal standard peak area. The peak detection limit was determined based on a signal-to-noise ratio of 3. Relative peak areas under the peak detection limit were imputed by K-nearest neighbor method. Putative metabolites were then assigned from HMT’s standard library and the Known-Unknown peak library based on *m*/*z* and MT with tolerances of ±10 ppm and ±0.5 min, respectively. If several peaks were assigned to the same candidate, the candidate was assigned a branch number.

### 4.4. Statistical Analysis

We compared clinical characteristics between responder and non-responder groups by chi-squared test for categorical variables. To explore metabolome distribution by groups, we performed principal component analysis with Pareto-scaled data using MetaboAnalyst 5.0 [39]. Then, metabolic features were hierarchically clustered by Ward method with Euclidean distance, following autoscale feature standardization. Significant metabolites between responders and non-responders at each timepoint (baseline, 6 months post-intervention, 18 months post-intervention) were determined by nonparametric Wilcoxon rank-sum test (false discovery rate adjusted *p*-value < 0.05, fold change > 1.2). Significant metabolites between baseline and 6 months post-intervention or baseline and 18 months post-intervention were determined by paired t-test (false discovery rate adjusted *p*-value < 0.05 and fold change > 1.2). With the significant metabolites, we identified enriched metabolite sets based on KEGG supported by MetaboAnalyst 5.0 and mapped the metabolites according to chemical and biochemical relationships by MetaMapp [40]. Metabolite networks were visualized with Cytoscape 3.8.2 [41].

## Figures and Tables

**Table 1:** Demographic characteristics of the study population.

		Responder (*n* = 20)	Non-Responder (*n* = 20)	*p*-Value ^1^
Age (years) ^2^		11.1 ± 2.1	11.0 ± 2.4	0.908
Sex (%)	Male	13 (65)	8 (40)	0.113
	Female	7 (35)	12 (60)	
Intervention type (%)	Exercise group	7 (35)	8 (40)	0.587
	Nutrition care group	9 (45)	6 (30)	
	Usual group	4 (20)	6 (30)	
BMI z-score	Baseline	3.04 ± 1.10	2.96 ± 0.92	0.791
	M06	2.83 ± 1.29	2.93 ± 0.94	0.781
	M18	2.07 ± 1.32	3.33 ± 0.94	0.001
Difference of BMI z-score	Baseline-M06	−0.21 ± 0.42	−0.03 ± 0.19	0.081
	Baseline-M18	−0.97 ± 0.44	0.38 ± 0.32	<0.001
AST	Baseline	27.8 ± 19.49	23.6 ± 7.13	0.371
	M18	23.55 ± 13.84	22.60 ± 10.63	0.809
ALT	Baseline	37.05 ± 43.05	22.45 ± 14.80	0.160
	M18	26.45 ± 28.53	27.10 ± 24.02	0.938
TG	Baseline	108.35 ± 49.74	88.10 ± 40.14	0.165
	M18	104.95 ± 40.59	94.15 ± 44.90	0.430
HDL cholesterol	Baseline	50.15 ± 12.03	52.40 ± 12.30	0.562
	M18	52.05 ± 11.51	53.50 ± 15.20	0.736
LDL cholesterol	Baseline	120.65 ± 22.89	108.70 ± 16.26	0.065
	M18	114.55 ± 24.75	113.20 ± 22.90	0.859
TG/HDL	Baseline	2.47 ± 1.75	1.84 ± 1.03	0.172
	M18	2.23 ± 1.25	2.01 ± 1.32	0.595

^1^ *p*-value by chi-square test for categorical variables and *t*-test for continuous variables. ^2^ Clinical data was shown as mean ± standard deviation.

## Data Availability

All data are contained within the article or supplementary material.

## Supplementary Materials

The following are available online at https://www.mdpi.com/article/10.3390/metabo12010027/s1, Figure S1: Individual changes in clinical parameters by weight-loss intervention (red, non-responders; blue, responders), Figure S2: Volcano plots show that no metabolites between responders and non-responders at baseline (**A**), 6 months post-intervention (**B**), and 18 months post-intervention (**C**) are significant (FDR adjusted *p*-value < 0.05 by Wilcoxon rank-sum test, fold change (responder/non-responder) > 1.2), Figure S3: A flowchart of the study population. Table S1: Significantly changed metabolites by weight-loss intervention (baseline vs. 6 months post-intervention or baseline vs. 18 months post-intervention, FDR adjusted-*p* value <0.05, fold change > 1.2), Table S2: KEGG-based metabolite set enrichment analysis of significantly changed metabolites by weight-loss intervention after 18 months.
