# Supplementary material for: Metabolomic Signatures for the Effects of Weight Loss Interventions on Severe Obesity in Children and Adolescents
Source: Metabolites. 2021 Dec 30;12(1):27. doi: 10.3390/metabo12010027 (PMC8778282; doi:10.3390/metabo12010027)
Supplement: Supplementary file 1 [file metabolites-12-00027-s001.zip › metabolites-1531541-SI.pdf]

metabolites

Supplemental Data

Metabolomic Signatures for the Effects of Weight Loss Interventions on Severe Obesity in Children and Adolescents

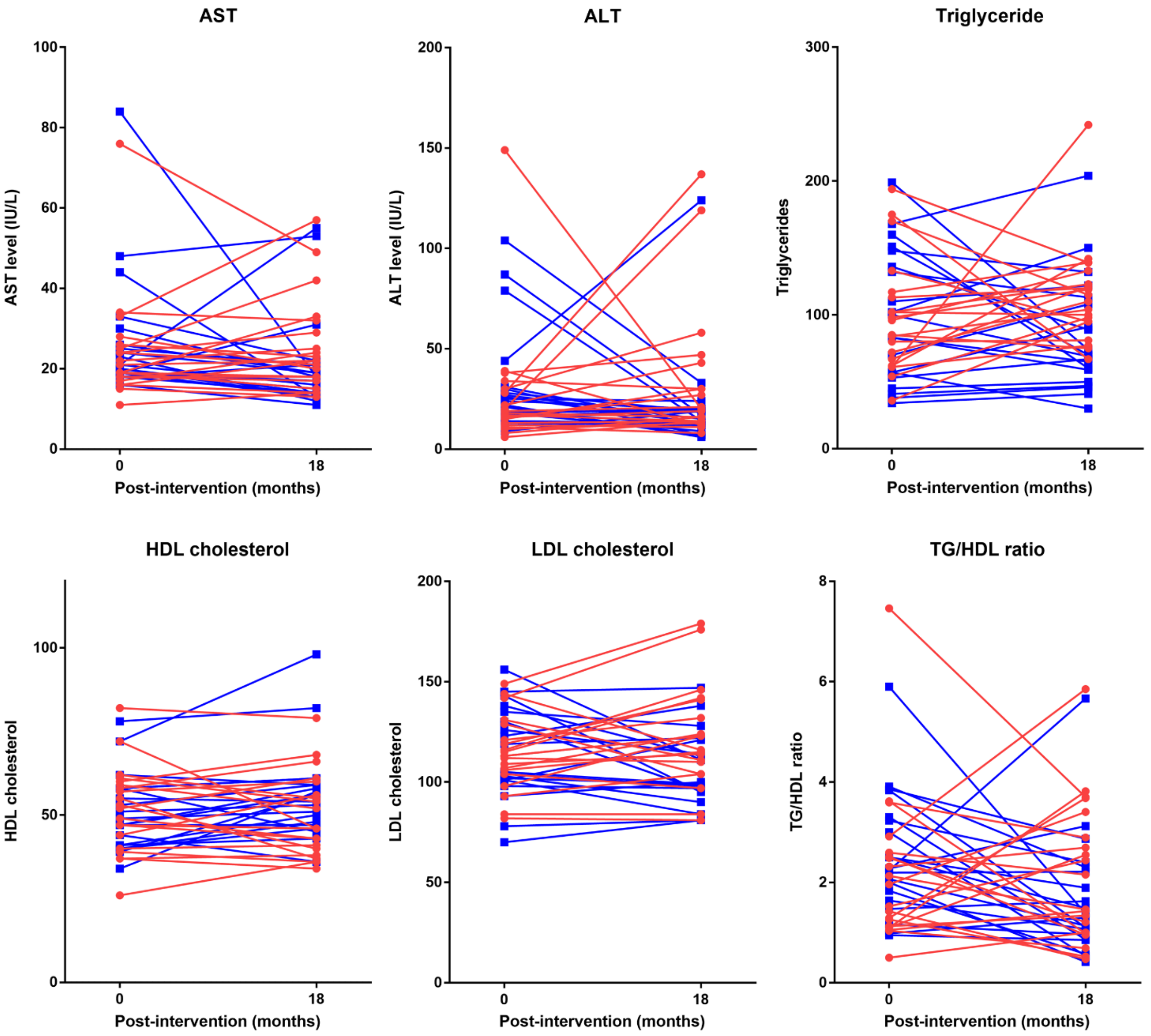

Figure S1. Individual changes in clinical parameters by weight-loss intervention (red, non-responders; blue, responders).

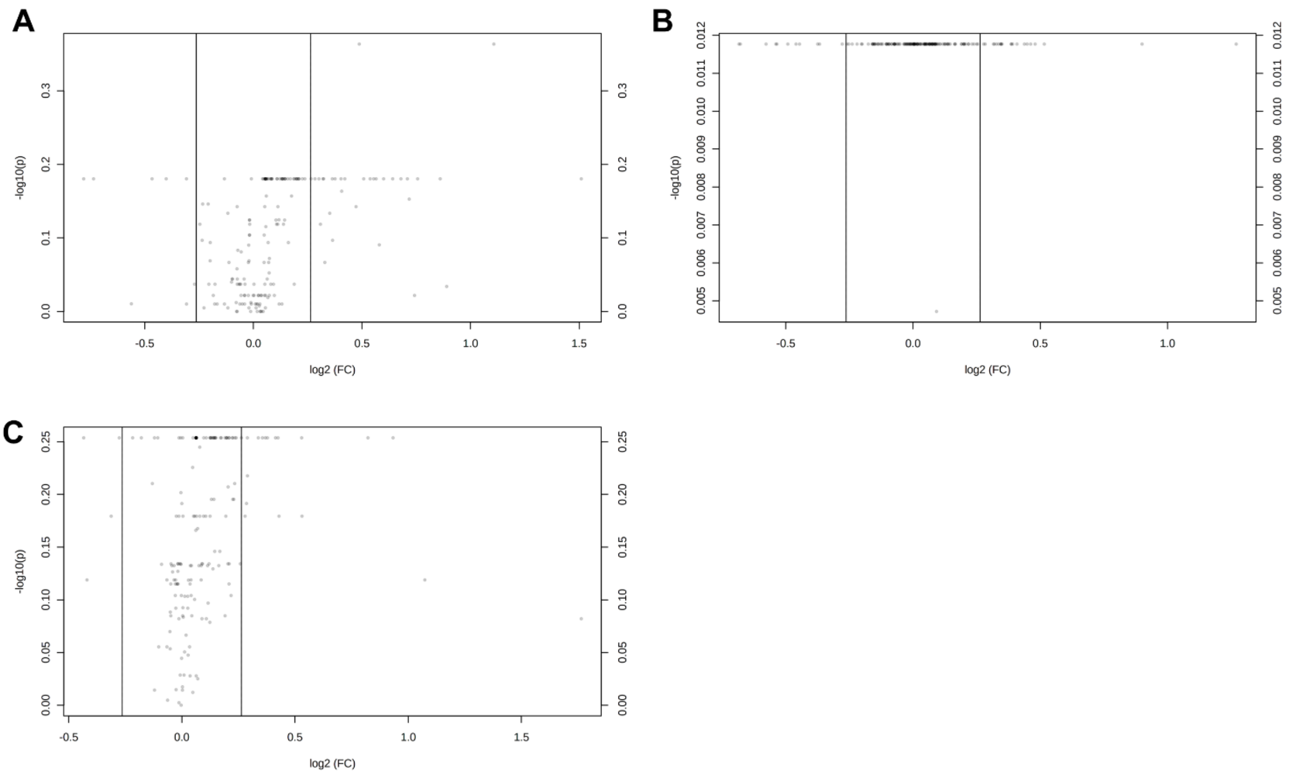

Figure S2. Volcano plots show that no metabolites between responder and non-responder at baseline (**A**), 6-month post-intervention (**B**), and 18-month post-intervention (**C**) is significant (FDR adjusted p-value < 0.05 by Wilcoxon rank sum test, fold change (responder/non-responder) > 1.2)

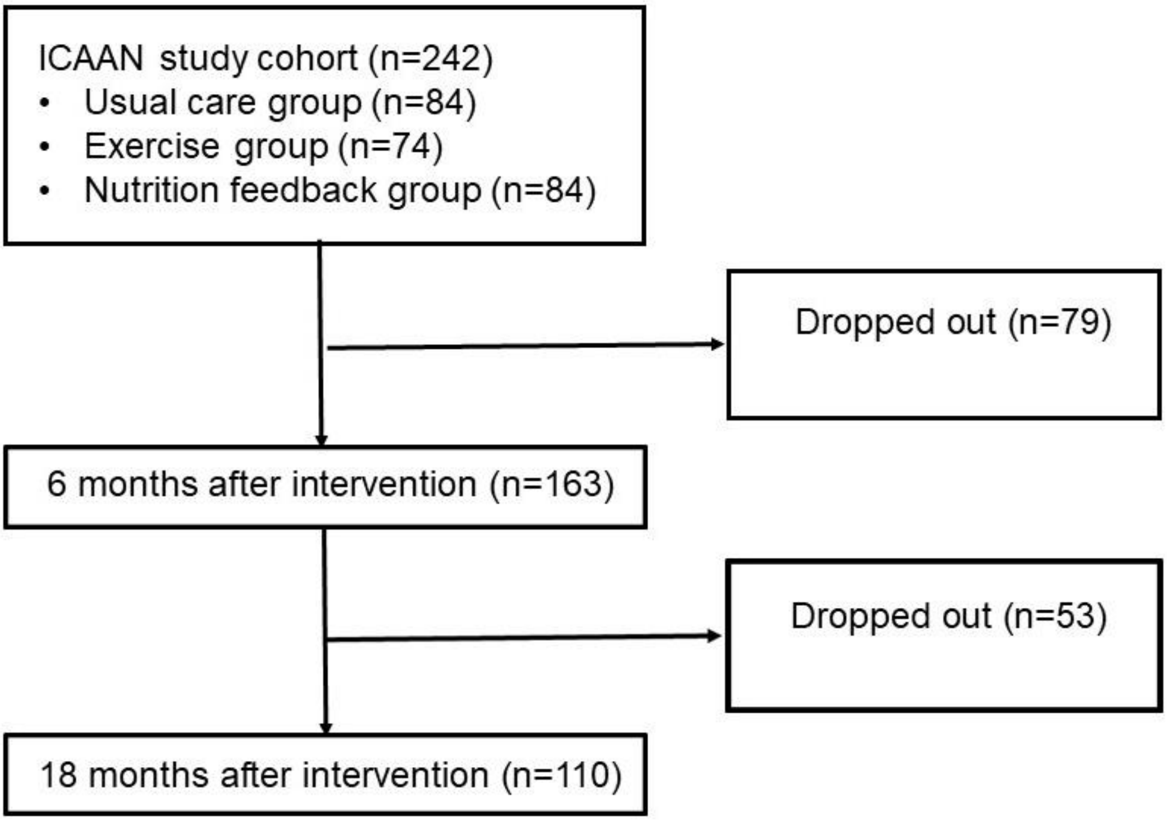

Figure S3. A flowchart of the study population.

Table S1. Significantly changed metabolites by weight-loss intervention (Baseline vs 6 months post-intervention or baseline vs 18 months post-intervention, FDR adjusted-p value <0.05, fold change > 1.2).

Metabolite	BL vs M06		BL vs M18	
	FDR adjusted p-value	FC (M06/BL)	FDR adjusted p-value	FC (M18/BL)
Alpha-ketoisovaleric acid	7.42E-01	0.92	**1.16E-23**	0.08
Methionine	3.28E-01	1.19	**7.15E-23**	12.64
Ketoleucine	2.14E-01	1.43	**1.93E-19**	10.57
Asparagine	**8.02E-03**	1.73	**9.65E-19**	5.55
Methionine sulfoxide	2.05E-01	0.86	**3.86E-18**	0.18
Glycerophosphocholine	**2.37E-02**	0.63	**5.06E-17**	0.13
N-Acetyllysine	**8.02E-03**	4.17	**6.80E-17**	37.84
Glutamine	**2.04E-04**	2.90	**5.55E-16**	8.60
S-Methylcysteine	1.75E-01	1.16	**1.62E-12**	2.12
O-Acetylcarnitine	**8.02E-03**	1.87	**1.36E-11**	3.47
1-Methyladenosine	2.28E-01	0.39	**3.59E-10**	0.02
4-Guanidinobutanoic acid	2.28E-01	2.77	**6.52E-10**	36.19
5-Oxoproline	1.03E-01	0.85	**7.12E-10**	0.47
Cystine	3.26E-01	0.77	**1.43E-09**	4.51
Serylglutamic acid	2.95E-01	2.04	**2.29E-09**	13.45
Glutamic acid	1.75E-01	0.94	**2.45E-09**	0.74
8-Hydroxyoctanoic acid	**2.15E-02**	4.10	**2.07E-08**	31.11
Valeric acid	1.74E-01	0.83	**2.07E-08**	0.53
Ornithine	4.52E-01	1.10	**3.38E-08**	0.62
Citramalic acid	8.47E-01	0.90	**6.36E-07**	3.02
Isocitric acid	7.42E-01	0.96	**2.03E-06**	0.73
Glucosamine	**8.02E-03**	0.70	**2.89E-06**	0.46
Aspartic acid	8.91E-01	1.08	**4.12E-06**	0.56
Uric acid	2.95E-01	1.11	**5.41E-06**	1.33
Lysine	7.35E-01	0.97	**7.47E-06**	0.82
3-Phosphoglyceric acid	2.73E-01	1.88	**3.92E-05**	6.27
Phosphorylcholine	1.06E-01	0.90	**8.03E-05**	0.77
Caproic acid	**6.42E-03**	0.66	**1.33E-04**	0.62
Adenosine monophosphate	7.54E-01	1.13	**2.25E-04**	0.58
Lactic acid	1.51E-01	1.17	**2.62E-04**	0.74
Alpha-Ketooctanoic acid	2.28E-01	2.33	**2.71E-04**	5.00
Sarcosine	2.39E-01	0.87	**3.69E-04**	0.70
Oxoglutaric acid	7.59E-01	1.01	**4.91E-04**	0.52
Galactaric acid	9.38E-01	0.94	**5.03E-04**	0.65
Succinic acid	6.54E-01	0.86	**5.21E-04**	0.61
Malic acid	5.62E-01	1.14	**6.56E-04**	0.76
Hydroxyoctanoic acid	8.26E-02	3.24	**7.38E-04**	4.67
Octanoylcarnitine	6.63E-02	1.23	**9.96E-04**	1.42
Arginine	**2.21E-02**	0.73	**1.54E-03**	0.70
Kyotorphin	**2.00E-03**	0.50	**1.68E-03**	0.78
Guanosine monophosphate	7.35E-01	1.59	**2.27E-03**	5.21
Threonic acid	9.38E-01	0.97	**4.79E-03**	0.83
Hydroxypropionic acid	9.38E-01	0.94	**5.18E-03**	0.48
Azelaic acid	1.54E-01	2.29	**7.27E-03**	2.83
N-Acetylornithine	**2.37E-02**	0.69	**9.63E-03**	0.75
N-Methylputrescine	7.08E-01	1.55	**1.28E-02**	3.64
Prostaglandin F2a	**1.09E-02**	0.77	**1.35E-02**	0.85
Hypotaurine	4.52E-01	0.88	**1.41E-02**	1.31
Hypoxanthine	7.35E-01	1.09	**4.39E-02**	1.45
Imidazolelactic acid	**1.60E-02**	3.51	8.53E-01	1.21

Table S2. KEGG-based metabolite set enrichment analysis of significantly changed metabolites by weight-loss intervention after 18 months.

Metabolite set	total	expected	hits	Enrichment ratio	Raw p	FDR
D-Glutamine and D-glutamate metabolism	6	0.189	3	15.9	**0.000551**	**0.0291**
Arginine biosynthesis	14	0.442	4	9.05	**0.000692**	**0.0291**
Alanine, aspartate and glutamate metabolism	28	0.883	4	4.53	**0.0102**	0.286
Citrate cycle (TCA cycle)	20	0.631	3	4.75	**0.023**	0.406
Valine, leucine and isoleucine biosynthesis	8	0.252	2	7.94	**0.0242**	0.406
Purine metabolism	65	2.05	5	2.44	0.0503	0.705
Butanoate metabolism	15	0.473	2	4.23	0.0788	0.945
Propanoate metabolism	23	0.725	2	2.76	0.162	1
Phosphonate and phosphinate metabolism	6	0.189	1	5.29	0.175	1
Nitrogen metabolism	6	0.189	1	5.29	0.175	1
Aminoacyl-tRNA biosynthesis	48	1.51	3	1.99	0.19	1
Taurine and hypotaurine metabolism	8	0.252	1	3.97	0.227	1
Glyoxylate and dicarboxylate metabolism	32	1.01	2	1.98	0.268	1
Glycine, serine and threonine metabolism	33	1.04	2	1.92	0.279	1
Cysteine and methionine metabolism	33	1.04	2	1.92	0.279	1
Glycerophospholipid metabolism	36	1.14	2	1.75	0.315	1
Arginine and proline metabolism	38	1.2	2	1.67	0.339	1
Valine, leucine and isoleucine degradation	40	1.26	2	1.59	0.362	1
Pantothenate and CoA biosynthesis	19	0.599	1	1.67	0.458	1
Ether lipid metabolism	20	0.631	1	1.58	0.475	1
beta-Alanine metabolism	21	0.662	1	1.51	0.492	1
Glycolysis / Gluconeogenesis	26	0.82	1	1.22	0.568	1
Glutathione metabolism	28	0.883	1	1.13	0.596	1
Amino sugar and nucleotide sugar metabolism	37	1.17	1	0.85	0.699	1
Pyrimidine metabolism	39	1.23	1	0.81	0.718	1

Bold values indicate raw p or FDR adjusted $p < 0.05$.
